# Emerging ethical issues regarding digital health data. On the World Medical Association Draft Declaration on Ethical Considerations Regarding Health Databases and Biobanks

**DOI:** 10.3325/cmj.2016.57.207

**Published:** 2016-04

**Authors:** Christine Aicardi, Lorenzo Del Savio, Edward S. Dove, Federica Lucivero, Niccolò Tempini, Barbara Prainsack

**Affiliations:** 1 King´s College London, London, UK; 2 Christian-Albrechts-Universität zu Kiel, Kiel, Germany; 3 University of Edinburgh, Edinburgh, UK; 4 University of Exeter, Exeter, UK

Throughout many parts of the world, biomedical research ethics is based on a core body of well-established norms, rules, and principles, including the Declaration of Helsinki, the Nuremberg Code, the Belmont Report, and the International Ethical Guidelines for Biomedical Research Involving Human Subjects (1). The overarching goal of these codifications is to protect people against harms arising from research, and from researchers experimenting on them without their knowledge and permission.

More recently, and because of the increasing technological capabilities for digital data production, storage, and analysis (such as web and ubiquitous computing, wearables, apps), new opportunities for conducting biomedical research have emerged. Data-intensive approaches in biomedicine, made possible by the increasing availability of digital health data and biobanks, are profoundly changing the ways in which research is conducted, and the role of research participants and health care (2-4). Physical contact between researchers and study participants is no longer needed and, once collected and stored, participants’ data remain available for potential reuse in further research for various purposes. With the growth of data-intensive approaches in biomedical research, a rich discussion on the saliency of moral considerations pertaining to the management of personal data in health databases and biobanks has unfolded (5).

## The World Medical Association (WMA) draft on Ethical Considerations Regarding Health Databases and Biobanks

Many regional, national, and international institutions that make policy on biomedical research ethics recognize this development. Some are revising their position on the ethics of data-driven biomedical research to keep pace with these transformative developments. In March 2015, for example, the WMA opened a public consultation on their “Draft on ethical considerations regarding health databases and biobanks.” The Declaration was available for public consultation until June 2015 (*http://www.wma.net/en/20activities/10ethics/15hdpublicconsult/).* It is now undergoing revision by a WMA working group in light of the public comments received. A definitive version is expected in 2016.

The WMA’s three-page draft Declaration is intended to complement the Declaration of Helsinki by providing “additional principles for the ethical use of data in Health Databases and human biological material in Biobanks” (Article 2). The Declaration defines “health databases” as systems for collecting, organizing, and storing health information, which enable the information to subsequently be retrieved in a structured manner; and “biobanks” as collections of biological material and associated data from different individuals (Article 3).

The Declaration is divided into three parts: a Preamble (Articles 1-12), a section on relevant ethical principles (Articles 13-22), and a section on governance (Articles 23-27). After defining “health databases” and “biobanks” as well as specifying their potential to accelerate biomedical research, the Declaration declares its remit to include “the use of health information beyond the individual care of patients” (Article 4). This means that health information used for research purposes is within the remit of the Declaration. Not included within its remit, however, are health databases and biobanks containing “fully anonymized or non-identifiable data” (Article 8).

The ethical principles that underpin research ethics policies typically seek to protect and enhance participants’ rights to privacy, confidentiality, and self-determination with respect to the disclosure of their information for research purposes. The draft Declaration indeed stresses the importance of respecting these rights, manifested, as it is commonly the case, in the duty to obtain the participant’s consent to have their identifiable information included in a health database or their biological material deposited in a biobank. Conditions that make informed consent possible on the side of the patient or participant include the need to receive clear details on the research modalities (eg, how participant’s data will be used, for what purpose, what privacy arrangements are in place) (Article 15). The WMA Draft Declaration also mentions participants’ right to request the correction of mistakes or omissions to their data (Article 16) and the right to withdraw consent for their identifiable information to remain included in a health database and their biological material to remain in a biobank (Article 17). These rights bear clear resonance with the recent data protection regulation reform in the European Union (6).

The WMA’s draft Declaration also considers the practices of blanket and open consent as ethically unacceptable. Conditional broad consent (Article 18) (ie, consent to future research studies), in contrast, is deemed ethically acceptable provided that: “during the consent process, all principle information about future use is provided, all relevant safeguards are secured, the use of health data or biological material is transparent, and if all use is explicitly approved by a research ethics committee.”

The draft Declaration calls for a “dedicated independent ethics committee” to approve the establishment of health databases and biobanks (Article 20) as well as to approve all use of data and human material and decide on the type of consent necessary, taking into consideration risks and benefits of the activity. As far as governance is concerned, it requires appropriate management and safeguards. For instance, adequate governance arrangements should be made concerning the purpose of the health database or biobank, the modalities of collection and access, the process to obtain consent, the length of time for storage, and the responsible individuals for governance and procedures for addressing enquires and complaints (Article 26).

Such is the brief background. While we support the codification of ethical principles for use of data in health databases and human biological material in biobanks, particularly given the pervasive use of digital health data, we find that the remit of the draft Declaration is unduly narrow and fails to offer meaningful advancement of the ethical principles stated in the Declaration of Helsinki. Changing practices in the collection and use of digital data require a revised framework and nomenclature regarding the norms, rules, and principles governing biomedical research. In the remainder of this article, we discuss five areas that ought to be taken into consideration in this process. These areas relate to shifts in health data-centric biomedical research that are relevant for governance and regulation (Table 1). Although our discussion takes the draft WMA Declaration as a reference point, our argument is applicable to the regulation of the collection and use of health data more broadly. By doing so, we also seek to contribute to scholarship on “knowledge landscapes,” a concept that acts as an analytical tool to map and scrutinize the heterogeneous sources, flows, and uses of e-health systems (7). We believe that such an integrated approach that foregrounds how knowledge landscapes are used and navigated when scrutinizing its ethical, regulatory, and social challenges, is much more timely and fruitful than anchoring our analysis in the domain in which a database is located, be it clinical, scientific, or commercial.

**Table 1:** Shifts in the collection and use of health data and their implications for biomedical research

Shift	What is the challenge?
Changing concept of “personal data”	Personal data are personal for a wider range of people than the individual from which they were collected or otherwise processed.
Limits of anonymization	Data are never fully anonymized in the sense that the re-identification individuals becomes impossible. New opportunities for data linkage and the integration of different data sets can make re-identification possible. Further, anonymization may not be the best means to protect and promote the interests of both researchers and participants.
Added pressures on consent procedures	When data are collected and stored for future use, it is impossible to anticipate all future uses and thus require fully informed and specific consent.
Transferability of health data to other domains (and vice versa)	Virtually any data set can be used to make health-relevant inferences pertaining to individuals (especially in the context of predictive analytics). Thus, also data that were not collected for health-relevant purposes can be used in a health-relevant way.
Risks associated with predictive analytics	It is very difficult, if not impossible, for individuals to know what data are used to make inferences and predictions about them. If data are used to harm them, or if inaccurate data are used, there are typically few options to rectify the harm/error or seek redress.

## What are personal health data? What is a health database/biobank?

The draft WMA Declaration makes repeated reference to individual-level health data. An aspect that the draft does not refer to explicitly is that health data increasingly travel between the clinical and non-clinical domains, and beyond. In the digital era, health databases can be linked with other types of databases, including those that do not originate in the clinic (eg, administrative and commercial databases), in order to extend the range of use of the data. In principle, no type and source of data are excluded from being used for health-related purposes (either in the clinic or for research) or in the commercial domain. Even web browsing data, the use of which is mostly unregulated, can offer important clues about one’s health (8,9). In this light, should we treat all data that can be mined for health-relevant purposes at a successive stage as “health data”? What about the metadata that databases also include? Moreover, in the context of increasing portability of health information, databases themselves might host data that were imported from elsewhere; what forms of consent would then apply when data sets converge? At stake are the boundaries of what counts as “data collection” as much as what counts as “health data.”

Given this definitional challenge, it is important to draw finer distinctions between different types of biobanks and health databases according to their mission, practices, uses, and the commercial stakes involved in them, especially along the dimensions outlined in Table 2.

**Table 2:** Descriptive dimensions of biobanks and health databases

Category	Questions to be asked
Content	What kind of data and/or materials are stored in the database/bank?
Personal data relationships	Can relevant demographic and familial relations be inferred directly from the data? (eg, genetic and genomic data sets, genealogical databases, etc.)
Intended uses of the data	Is the primary intended use of the data biomedical research, or something else? Does data use aim to create public benefits? What are the commercial stakes and interests?
Security standards	What are the security standards (eg, cloud-based databases with data located at participating institutions; data centralized in data safe havens; access control policy)?
Actual uses of the data	Who is using the data, and for what purposes?
Mode of governance	Who can populate the database? Who can access data? Who decides on access? Is there transparency on these decisions, and on how these decisions are made?

## Personal data are personal for more than one person

The draft WMA Declaration’s emphasis on rights (eg, to privacy) to the detriment of duties (eg, to communicate actionable findings that impact on the health of others) may push to the background other important moral and ethical concerns that arise in the context of data-rich biomedical research. One such concern emerges from the fact that “personal” and “individual” data are not synonymous, although they are often treated as such (10-12). Biological information often discloses something both about the person who donated the data or sample and biologically related (or even unrelated) others. For instance, much genetic data provide information about more than just one individual (13). Consequently, both harms and benefits, and the appropriate balance and relationship between rights and duties, need to be considered. In particular, the notion that rights to privacy, confidentiality, and self-determination entitle an individual to exercise control over the use and disclosure of information concerning her- or himself in every instance, especially in the case of genetic information, ought to be challenged in light of this broader understanding of the personal nature of data. Should an individual person have an unfettered “right” to control the communication of an actionable research finding to a genetic relative that may have implications for him or her?

We may also imagine that through a number of people each giving access to their own individual records for research, or even sharing their data publicly, it may be possible to establish a probabilistic correlation of clinical relevance between two characteristics A and B (eg, a biomarker and late onset disease). The sheer volume of data and the relative ease with which digital data can be mined would make this possible. For any individual exhibiting characteristic A, the hypothesis of them also possessing or developing characteristic B could lead to tangible implications, even if the quality of the data and the robustness of the correlation were never verified. This raises the question of how such practices can be regulated. The European Union’s forthcoming General Data Protection Regulation will give important additional rights to data subjects that will make it more difficult for corporations to use data without the knowledge of the data subject, and will make it easier for people to seek redress (6). It remains to be seen, however, whether large corporations with pockets deep enough to tolerate pecuniary penalties will comply with the new rules, and how they will be enforced (14,15). Further, let us imagine that one of the correlated characteristics was associated to an observable (physical or behavioral) trait and that some linkage could subsequently be made between the trait and the increased likelihood of a specific condition. To use a well-known example, BRCA mutations increase the risk of breast and ovarian cancer manifold. Similarly cancer risk increases with consumption of processed meat or alcohol. It is likely that other correlations will be discovered in the future. The issue has been raised by scientists working on medical data analytics in a large collaborative research project funded by the European Commission (16), in relation to the use of unsupervised learning techniques in medical data mining: What happens when these techniques yield such correlations, warranted or not, that are likely to result in social discrimination – positive or negative – for the individuals exhibiting this particular trait?

## Anonymization is not forever

Article 8 of the draft WMA Declaration states that health databases and biobanks that “exclusively contain fully anonymised and non-identifiable data and biological material” are excluded from the remit of the Declaration. The notion that “full anonymization” offers the best protection and promotion of participants’ interests needs to be reconsidered. Not only is the anonymity of data and material highly context-dependent, but data and material that are anonymized today may no longer be anonymous in the context of tomorrow’s technologies and data resources. Whatever is contained in a health database or a biobank may be anonymized and non-identifiable at the time it is set up, but this may not remain so over time, especially when data from the database or biobank are linked with other data sets. The re-identifiability of the information in a database or biobank is relative and contingent, and therefore needs to be reconsidered regularly. Exemptions from research ethics requirements for supposedly anonymized data gives the false and dangerous impression that anonymized data are inherently less prone to re-identification. Policies should be very clear about ethical concerns about data and biological materials continuing even after anonymization, but also that anonymization is not a process that necessarily promotes the interests of both researchers and participants at all times. Sophisticated information security designs at both the technological and organizational levels can go a long way in protecting participants’ identity while ensuring long-term consistency of the research infrastructure, but whether, and under what circumstances, should and could anonymization be reversible is a very complex terrain that must be explored contextually in every project.

It is also worth emphasizing yet again that the term “anonymous” must be distinguished from “anonymized”. Anonymization is a process performed on identifiable data or material, which makes the data or material no longer identifiable. This concept is categorically distinct from “anonymous,” which signifies a status of data or material, namely that which never was identifiable at the origin. This is a crucial distinction from a data protection law perspective, because it means that the processing of personal data for the purposes of achieving anonymization (ie, the rendering of personal data to an anonymized state) remains subject to data protection laws. That is, prior to the completion of this process of anonymization, the data are still “personal” in the way that data protection laws define the term (17). It must be noted, however, that the distinction is frequently challenged in a time when distributed and automated data collection and record linking are increasingly available.

## Varieties of consent

Informed consent is a long-standing pillar of ethical research. However, emerging forms of science that focus on data analytics rather than bodily intervention encourage a rebalancing of personal autonomy and societal interests. It can be the case that other forms of consent may render research ethical in databased biomedical science. The “classic” form of specific consent (ie, consent to a specific study) must be contrasted with non-specific consent. The latter includes open, broad, and blanket consent, on which a vast literature has emerged (18-21). “Open” consent is “consent to unrestricted redisclosure of data originating from a confidential relationship, namely (...) health records, and to unrestricted disclosure of information that emerges from any future research on (...) genotype-phenotype data set, the information content of which cannot be predicted,” with no promise of anonymity (22). “Blanket” consent puts no restriction to scope and duration of consent, whereas “broad” consent restricts use of personal data to broad areas of research, eg, biomedical research. In general, obtaining specific informed consent need not be an absolute requirement in the context of data-centric research, as forms of non-specific consent may be ethical even if they are not universally regarded as “truly informed” consent. Moreover, in some circumstances, anonymous or anonymized data that were collected for a different purpose may be used legally and ethically for research purposes in some jurisdictions, including when it is not reasonably possible to re-contact the data donor for consent. On this basis, large databanks that have been constructed by aggregating and linking data sets collected in the context of routine and administrative services can now support a variety of different kinds of research projects.

## Predictive analytics, commercial uses, private partnerships

Personal data are increasingly used by commercial companies, notably in the context of consumer scoring. This creates various types of risks to individuals and groups and in varying orders of magnitude. They are relevant to the governance of biobanks and data sets as the ownership of data and samples stored in a biobank can change and give rise to uses of data that were not intended by the biobank’s initial mission. The issue of funding of health databases and biobanks, and the related concern of sustainability, ie, what happens upon the termination or winding down of a database or biobank, are of crucial importance in this respect (23). It must furthermore be noted that the boundaries between health care, commercial, and research purposes are increasingly blurred. This is evidenced, for example, by the fact that a private company selling personal genomics services (23andMe) received NIH funding to build survey tools, expand its gene database, and use its stores of genetic data for research projects (24,25). Another example is that a philanthropic foundation established a $20 million endowment at Harvard Business School “to find ways to accelerate breakthroughs and advance commercialization of precision medicine by harnessing the energy and ideas of the medical, science and entrepreneurial communities in the city” (26). More easily than ever before, data generated for one kind of purpose and services (eg, marketing) can be repurposed for other kinds of projects and services (eg, health and social care).

In the everyday operation of a health data project, there are governance issues associated with the need to provide up-to-date and relevant information as these projects develop and ramify through partnerships. Digital technologies can offer relatively inexpensive opportunities for transparent and inclusive communication, but solutions will have to be contextual to the project.

## Discussion

The ethical guidelines for health databases and biobanks outlined in the WMA’s draft Declaration define not only rights of participants, but also what counts as data gathering, linking and handling, scientific research, and even medical practice. As such, the draft Declaration does not simply specify the conditions that need to be met for research to be considered ethical, but it also creates expectations, imagines valuable futures, and defines what desirable and undesirable scenarios would be. Although the Declaration of Helsinki was conceived as a deontological professional code for physicians, these stakeholders should welcome the opportunity to expand ethical reflections to new issues arising in biomedical research, namely that of digital health data. The challenge ahead is not so much that of extending existing ethical principles and reflecting on how they play out in a changing landscape of data collection and use. Instead, along with methodological changes, the management of health databases and biobanks is accompanied by changes in the social, economic, and moral order, which require a new language in which we frame and address the ethical challenges arising. The concept of “knowledge landscapes” would be a useful tool toward this goal, as it enables us to identify interrelated areas of information, knowledge, and associated narratives and practices, rather than constraining our analysis by existing nomenclatures and categories that separate traditional “types” of databases and domains of use (7).

## Conclusions

On the basis of the broader approach to the analysis of knowledge production underpinned by the notion of “knowledge landscapes,” we have argued that digital health data require rethinking norms, categories, and nomenclatures governing biomedical research. In particular, we have identified five shifts in the collection and use of digital data that are of immediate relevance to the regulation of such research (Table 1).

These shifts call for a broadened ethical discussion pertaining to digital health data. Ethical guidelines and policies need to be understood as having a socially constructive role in the future directions of biomedical research. The making of such policies is an act of social imagination that encompasses the full array of actors in the biomedical research ecosystem: health professionals, regulators, participants, and publics. As we are all affected, we are all encouraged to deliberate on the ethical issues at play.

## 

**Table:** 
